# Indications and Morbidity of Reoperative Thyroid Surgeries in a Military Hospital of Senegal

**DOI:** 10.1155/2017/4045617

**Published:** 2017-09-11

**Authors:** Abdou Sy, Eric Joël Regonne, Aminata Fofana, Yves Diandy, Malick Ndiaye

**Affiliations:** ^1^ENT Department, Ouakam Military Hospital, Dakar, Senegal; ^2^ENT Department, Children's Hospital of Diamniadio, Dakar, Senegal

## Abstract

**Objectives:**

To describe reoperative thyroid surgeries in our department.

**Study Design:**

Retrospective cross-sectional and descriptive study at the Ouakam Military Hospital in Dakar (Senegal), over a period of eight and a half years.

**Methods:**

The study involved all records of patients who had a reoperative thyroidectomy regardless of the indication and time of the second surgery. Parameters evaluated for first and reoperative surgery were time interval between the two surgeries, operative indications, surgical procedures, intraoperative findings, pathological examination, and morbidity.

**Results:**

30 records of patients were selected out of a total of 698 thyroidectomies (4.3%). Thyroid cancers diagnosed on first surgical specimens were the first indications of reoperations (46.67%) followed by neck hematoma (20%). Completion thyroidectomy with a prophylactic central lymph nodes dissection was the most performed surgical procedure (43.33%) followed by haemostasis (20%). During reoperation, we found active bleeding (20%), textiloma (6.67%), and fourth branchial cleft fistula (3.33%). The morbidity accounted for 10%: lymphorrhea, permanent hypocalcemia, and permanent recurrent nerve palsy, in one case, respectively. There were no statistically significant differences between the morbidity in patients reoperated on and the one for patients operated on once.

**Conclusion:**

We did not find an increased risk of postoperative morbidity after reintervention.

## 1. Introduction

Reoperative thyroidectomy is a rare surgical procedure. Its frequency in the literature varies from 4.7 to 7.5% [1–4]. Its main indications are thyroid cancers diagnosed on specimen of lobectomy with isthmusectomy or subtotal thyroidectomy, recurrence of thyroid cancers and benign goitres, and neck hematoma. It is a surgical procedure which exposes the patient to a risk of recurrent laryngeal nerve and parathyroid injury, due to fibrosis and anatomical changes, the consequences of the previous intervention on the thyroid bed. Some authors find that the incidence of postoperative complications after reoperative thyroidectomy is significantly greater than after a first thyroidectomy [4, 5], while others find that there are none [6, 7].

The objectives of our study were to describe the indications for reoperative thyroid surgeries, to determine the incidence of postoperative complications, and to compare them with thyroidectomies performed during the same period in our department.

## 2. Materials and Methods

We carried out a retrospective cross-sectional and descriptive study at the Ouakam Military Hospital (HMO) in Dakar, Senegal, over a period of eight and a half years (from July 1, 2004, to December 31, 2012). It involved all records of patients who had a reoperative thyroidectomy regardless of the indication and time of the second surgery.

We defined reoperative thyroidectomy as any surgery on the thyroid bed that required a return to the operating room for reoperation either to complete the first surgery (lobectomy, completion thyroidectomy, or lymph node dissection of the central compartment) or in relation to a complication related to the first surgery (haemorrhage, textiloma, infection, etc.) and whatever the time between the two interventions.

Direct or indirect laryngoscopy was performed for all patients prior to surgical reoperation. The reoperation was performed under general anesthesia for all patients, taking again the same skin incision Kocher type. We did not use intraoperative monitoring of recurrent nerves because it is not available in Senegal.

A direct or indirect laryngoscopy was performed systematically for all patients on the second postoperative day. The dosage of serum calcium was systematic in all postoperative patients. It was repeated the next day (Day 2) if the first serum calcium was low.

We defined hypocalcaemia as a serum calcium below 2.20 mmol/L or 88 mg/L. A complication was defined as permanent if it lasted more than six (06) months.

The studied variables were as follows:

- For the first surgery: operative indication, surgical procedure, and results of the pathological examination of the surgical specimen.
- For reoperations: the time between the two procedures, operative indication, surgical procedure, intraoperative findings, and results of the pathological examination of the surgical specimen and morbidity.

We compared the frequencies of complications after reoperative thyroidectomy (recurrent paralysis and hypoparathyroidism) with those occurring after the thyroidectomies performed in our department during the study period. The data was recorded and analyzed using Microsoft Excel 2013. We used the Fisher Exact Test to compare the qualitative variables. A value of *p* < 0.05 was considered significant.

## 3. Results

A total of 30 records of patients were selected. During the same period, 698 thyroidectomies were performed. Reoperative thyroidectomy surgery accounted for 4.3% of thyroidectomies.

### 3.1. Sociodemographic Data

The average age of patients was 38 years. The extremes were 8 and 65 years old. There were 27 women and 3 men, a sex ratio of 0.1. For 26 patients, the first thyroidectomy was performed in our structure and in 4 cases it was done in other hospitals.

### 3.2. The First Thyroidectomy

#### 3.2.1. Surgical Indications and Surgical Procedure

The indications were multinodular goitre in 23 cases (76.67%), Graves' disease in 6 cases (20%), and repeated suppurated thyroiditis in 1 case (3.33%).  Table 1 presents our surgical attitude according to the indications given during the first thyroidectomy. Lobectomy with isthmusectomy was the most performed surgical procedure (53.33%).

#### 3.2.2. Pathological Examination of the Surgical Specimens

Pathological findings were available for 21 patients (70%). They showed

- papillary carcinoma in 9 cases (42.86%);
- benign thyroid nodules in 5 cases (23.81%);
- vesicular carcinoma in 3 cases (14.29%);
- Graves' disease in 2 cases (9.52%);
- medullary carcinoma in 1 case (4.76%);
- Graves' disease associated with a suspicion of malignancy in 1 case (4.76%).

### 3.3. The Reoperative Thyroid Surgery

The average time between the two surgeries was 19 months with extremes ranging from 2 hours to 20 years. No patients reoperated on had a complication after the first surgery.

#### 3.3.1. The Surgical Indications

Thyroid cancers diagnosed on first surgical specimens were the first indications of reoperations (46.67%) followed by neck hematoma (20%) (Table 2).

#### 3.3.2. The Surgical Procedure

Completion thyroidectomy with a prophylactic central lymph nodes dissection was the most performed surgical procedure (43.33%) followed by haemostasis (20%). A resection of a fourth branchial cleft fistula had been performed, which had been revealed as a left suppurated thyroiditis, and for which the patient had undergone first a left lobectomy with isthmusectomy. Prophylactic lymph nodes dissection was performed for all cases of thyroid cancer (Table 2).

#### 3.3.3. Intraoperative Findings

Table 3 presents the findings made during the operation. Active bleeding was found in 6 cases. For 4 patients, it came from a branch of the superior thyroid artery and for one patient from a branch of the middle thyroid artery and diffuse oozing was noted in the last patient. Concerning the patient with a central lymph nodes dissection alone, we did not discover enlarged lymph nodes during operation. We performed a lymph node dissection because the pathological examination of the surgical specimen showed thyroid cancer.

#### 3.3.4. Pathological Examination of Surgical Specimens

A pathological examination of surgical specimen was performed for patients whom we discovered during exploration: a thyroid lobe, a superior polar remnant, and an enlargement of the pyramidal lobe (20 cases). Pathological examinations were available for 14 of them. They showed

- a benign multinodular goitre in 4 cases;
- thyroid adenoma in 4 cases;
- a normal thyroid parenchyma in 3 cases;
- Graves' disease in 2 cases;
- a revamped thyroid tissue with no tumor in 1 case.

#### 3.3.5. Follow-Up

The postoperative period was uneventful for 27 patients. Three patients (10%) had complications. These were lymphorrhea, permanent hypocalcaemia, and permanent recurrent nerve palsy, in one case, respectively (3.3%, resp.). Of the 668 patients with a single thyroidectomy, 5 had permanent recurrent paralysis (0.7%) and 3 had permanent hypocalcaemia (0.4%). There were no statistically significant differences between the incidence of recurrent paralysis in patients reoperated on and in patients operated on only once (3.3% versus 0.7%, *p* = 0.23). There were no statistically significant differences between the incidence of hypocalcaemia in patients reoperated on and the one for patients operated on once (3.3% versus 0.4%, *p* = 0.16). The average follow-up period was 2.5 years with extremes of 3 months and 7 years. Operative mortality was zero.

## 4. Discussion

In the literature, the frequency of reoperative thyroidectomy is variously estimated. It depends on the methodology used for the recruitment of the subjects and the surgical indications retained for the study. As a result, it is difficult to make comparisons between the various published series. However, in the literature, this frequency varies from 4.7 to 7.5% [1–4].

There are many indications of thyroidectomy reoperations. The main indications in the literature are thyroid cancers diagnosed after lobectomy with isthmusectomy or subtotal thyroidectomy, recurrences of thyroid cancers and benign goitres, and neck hematoma. Thyroid cancers were the first indication of reoperation in our series. In the literature, the management of a thyroid nodule and the management of differentiated thyroid cancers are well codified [8, 9]. In our context, the lack of intraoperative frozen section examination and the weak realization of fine-needle aspiration cytology make these cancers most often diagnosed on a specimen of lobectomy with isthmusectomy [10]. When the diagnosis of cancer is confirmed at the pathological examination, a lymph node dissection is envisaged. Its indications take into account the condition of lymph nodes areas to preoperative imaging and during intraoperative exploration. This is a controversial subject in the literature [8]. We have a more aggressive attitude; it consists of systematically performing a completion thyroidectomy with a prophylactic central lymph nodes dissection. This attitude is justified by the fact that, in our context of work, it is difficult to monitor patients; they are generally lost for follow-up.

One of the worst complications of thyroid surgery is hematoma. It may occur within 48 hours after thyroidectomy, and 20 to 87.5% of the hematomas are diagnosed before 8 hours [11–14]. In our practice, we do not perform outpatient thyroidectomy as advocated by some authors [15] and prefer to be more cautious by hospitalizing patients for 48 hours in order to observe and manage any complication. According to the literature, after a thyroidectomy, 0.3 to 1.16% of patients have a secondary surgery for cervical hematoma [11–14]. It complicates 0.7% of thyroidectomies in our department [16]. For Shaha and Jaffe [14], the main cause of hematomas was the failure of ligatures from the major vessels of the thyroid gland. For Lee et al. [12], 50% of the hematomas came from the main vessels of the thyroid bed and 60% of them were a branch of the superior thyroid artery. In our series, 4 cases of hematoma out of 6 (66.67%) originated from bleeding from a branch of the superior thyroid artery. Clinically, hematoma manifests as respiratory distress with dyspnea and/or stridor, dysphagia, neck pain, cervical swelling, haemorrhage or bruising, and in extreme cases cardiac arrest [12, 13]. This is why it is recommended that the patient be closely monitored and listened to when he/she is in the recovery room or in hospital room. When these signs occur, the operative wound should be reopened, if necessary at the patient's bed, and an exploration should be performed in the operating room under general anesthesia. Sometimes an emergency tracheotomy is performed in case of severe respiratory distress [11–13], as it was the case for one of our patients. In our practice, the muscles of the thyroid bed are not hermetically closed and a “safety triangle” is left at the bottom of the white line at the time of closure. To limit the risk of bleeding, we recommend

- performing a median aponeurotomy on the white line and not an opening through the subhyoid muscles;
- performing meticulous haemostasis (cauterization and ligatures) at each stage of the thyroidectomy, the systematic double ligation of the upper thyroid arteries, and avoiding damage to the anterior jugular veins at the time of the cervical approach.

In order to see bleeding points, some authors suggest increasing the venous pressure, under general anesthesia, by the Valsalva maneuver or by placing the patient on Trendelenburg position (decubitus at an angle of 30°) before closing the operative wound [12, 13]. It is a technique that is not common in our daily practice.

In the literature, recurrence of benign multinodular goitre after subtotal thyroidectomy varies from 2.5 to 42%, after 5 to 30 years of follow-up [5]. This could be due to the development of new nodules in the thyroid gland or the growth of macroscopic or microscopic residual nodules that were unnoticed during the first surgical procedure [17]. This is why it is important to always palpate the contralateral lobe when a lobectomy is indicated. This is what we do in our practice. Some authors prefer to perform a partial excision after a recurrence of benign nodular goitre [3]. We have a pragmatic attitude, which consists in performing a thyroid totalization in case of a recurrence. This makes it possible to eliminate the risk of recurrence, thus avoiding a new reoperation, which increases the postoperative morbidity.

Postoperative infection is a rare complication of thyroidectomy. In the literature, its incidence varies from 0.36 to 3% [18, 19]. It was the indication of reoperations in three of our patients. For two of them, we discovered during the surgical exploration a textiloma. This involves an accidental retention of one or more gauze in the surgical site during a surgical procedure. Textiloma may be revealed as a wound healing delay, a fistula with a pus production at the level of the incision, or an abscess [20]. It is a complication of the thyroidectomy rarely found in the literature probably for medicolegal reasons; surgeons are reluctant to report them. The authors generally report clinical cases [20, 21].

The morbidity varies from one author to another, depending on whether it is about completion thyroidectomy or central lymph node dissection for thyroid cancers, or lobectomy after recurrence of a benign goitre. The fibrosis following the first procedure and the anatomical modifications make it more difficult to surgically approach the thyroid bed. Thus, it seems more difficult to spot laryngeal recurrent nerves and parathyroid glands. In the literature, reoperative thyroidectomies are performed with low morbidity [4]. We did not find any significant differences in complications between reoperative thyroidectomies and first surgeries. Our results corroborate those of Rafferty et al. [7] and Erdem et al. [6]. On the other hand, Menegaux et al. [4] in France found that the incidence of postoperative complications in patients reoperated on was significantly higher (10.4% versus 3.5%). Moalem et al. [5] in 2008, in a systematic review of multinodular goitre recurrences, found that patients reoperated on had more permanent recurrent nerve palsy and permanent hypoparathyroidism than those with a single surgery. It seems that the experience of the centers where the patients are operated on is a factor favoring the reduction of the risk of postoperative complications after a reoperation [2, 4, 6]. Hypoparathyroidism and recurrent nerve palsy are the main specific complications of thyroid surgery. In the literature, after a reoperative thyroidectomy, the complications are as follows:

- Transient recurrent nerve palsy: 0 to 8.5% [1, 4, 22–25]; permanent recurrent nerve palsy: 0 to 3.5% [1, 4, 6, 23–25].
- Transient hypoparathyroidism: 1 to 36.4% [1, 3, 4, 23–25]; permanent hypoparathyroidism: 1.1 to 4.2% [1, 4, 6, 23–25].
- Haemorrhage: 1.3 to 2.5% [1, 24]; hematoma: 2.5% [4].
- Infection of the operative wound: 0.5 to 3.8% [4, 24].

In order to reduce the risk of complications after a possible reoperation, some surgeons propose not to explore the contralateral side during a first surgery when a lobectomy is indicated [23]. This attitude requires, beforehand, to have well performing diagnostic means, especially in the field of medical imaging, in order to be sure that the side that is not to operate on is free from any pathology. However, they are often lacking in our work context, which imposes an almost systematic exploration of the contralateral lobe when a lobectomy or lobectomy with isthmusectomy is indicated. In the literature, the use of a monitoring of recurrent nerves is not of a great contribution in reducing the risk of lesion of these nerves during a reoperative thyroidectomy [2]. Prokopakis et al. [22] in 2013 in Greece showed it in their study. Indeed, the intraoperative monitoring of the recurrent nerves makes it possible to confirm their presence but does not allow giving their precise localization. Thus, the intraoperative visual identification of the recurrent nerves and the experience of the surgeon are the best pledges to avoid a lesion of these nerves.

*Limitations*. Although we did not find an increased risk of postoperative morbidity after reintervention compared with the first surgery, this may be due to the limited number of the patients who underwent reintervention.

## 5. Conclusion

Reoperative thyroidectomies are rarely performed. They have risks, even low, of recurrent and parathyroid morbidity. We did not find an increased risk of postoperative morbidity after reintervention. When performed by experienced surgeons, these risks are reduced. When this surgery is indicated, careful dissection and hemostasis should be performed to reduce morbidity.

## Figures and Tables

**Table 1:** Surgical attitude according to the indications during the first thyroidectomy.

Surgical procedure	Indication			
	Multinodular goitre	Graves' disease	Thyroiditis	Total
Lobectomy with isthmusectomy	15	0	1	16
Total thyroidectomy	3	4	0	7
Subtotal thyroidectomy	5	2	0	7
Total	23	6	1	30

**Table 2:** Indications of reoperative thyroidectomies with surgical procedure performed at the first surgery and the reoperation.

Indications	First surgery	Reoperation	Number
Thyroid cancer	Lobectomy with isthmusectomy	Completion + lymph node dissection	9
	Subtotal thyroidectomy	Completion + lymph node dissection	4
	Total thyroidectomy	Lymph node dissection	1
Neck hematoma	Total thyroidectomy	Haemostasis	5
	Lobectomy with isthmusectomy	Haemostasis + tracheotomy	1
Recurrence of benign nodular goitre	Lobectomy with isthmusectomy	Lobectomy	4
	Subtotal thyroidectomy	Totalization	1
Infection	Lobectomy with isthmusectomy	Exploration	1
		Fistula resection	1
	Total thyroidectomy	Exploration	1
Persistent hyperthyroidism	Subtotal thyroidectomy	Totalization	1
Recurrence of hyperthyroidism	Subtotal thyroidectomy	Totalization	1

**Table 3:** Distribution of patients according to intraoperative findings.

Intraoperative findings	Number	Percentage (%)
Single thyroid lobe	12	40
Upper polar remnant	6	20
Haemorrhage	6	20
Pyramidal lobe enlargement	2	6.67
Textiloma	2	6.67
Fourth branchial cleft fistula	1	3.33
Empty thyroid bed	1	3.33

## References

[1] Pelizzo M. R., Variolo M., Bernardi C. (2014). Complications in thyroid resurgery: A single institutional experience on 233 patients from a whole series of 4,752 homogeneously treated patients. *Endocrine*.

[2] Tresallet C., Chigot J. P., Menegaux F. (2006). Comment prévenir la morbidité récurrentielle en chirurgie thyroïdienne?. *Annales de Chirurgie*.

[3] Malki HO El., Mohsine R., El Mazouz S. (2002). Les réinterventions après thyroïdectomie pour goitre. *Ann Endocrinol*.

[4] Menegaux F., Turpin G., Dahman M. (1999). Secondary thyroidectomy in patients with prior thyroid surgery for benign disease: A study of 203 cases. *Surgery*.

[5] Moalem J., Suh I., Duh Q.-Y. (2008). Treatment and prevention of recurrence of multinodular goiter: An evidence-based review of the literature. *World Journal of Surgery*.

[6] Erdem E., Gülçelik M. A., Kuru B., Alagöl H. (2003). Comparison of completion thyroidectomy and primary surgery for differentiated thyroid carcinoma. *European Journal of Surgical Oncology*.

[7] Rafferty M. A., Goldstein D. P., Rotstein L. (2007). Completion Thyroidectomy Versus Total Thyroidectomy: Is There a Difference in Complication Rates? An Analysis of 350 Patients. *Journal of the American College of Surgeons*.

[8] Herry J. (2008). Prise en charge des cancers papillaires et vésiculaires de la thyroïde. *Médecine Nucléaire*.

[9] Haugen B. R., Alexander E. K., Bible K. C. (2016). 2015 American Thyroid Association Management Guidelines for adult patients with thyroid nodules and differentiated thyroid cancer: The American Thyroid Association Guidelines Task Force on thyroid nodules and differentiated thyroid cancer. *Thyroid*.

[10] Deguenonvo R. E. A., Diouf-Ba M. S., Dial M. C. (2016). Surgical Management of Differentiated Thyroid Carcinomas: Experience in a Low-Income Country. *Prensa Med Argent*.

[11] Abbas G., Dubner S., Heller K. S. (2001). Re-operation for bleeding after thyroidectomy and parathyroidectomy. *Head and Neck*.

[12] Lee H. S., Lee B. J., Kim S. W. (2009). Patterns of post-thyroidectomy hemorrhage. *Clinical and Experimental Otorhinolaryngology*.

[13] Burkey S. H., Van Heerden J. A., Thompson G. B., Grant C. S., Schleck C. D., Farley D. R. (2001). Reexploration for symptomatic hematomas after cervical exploration. *Surgery*.

[14] Shaha A. R., Jaffe B. M. (1994). Practical management of post‐thyroidectomy hematoma. *Journal of Surgical Oncology*.

[15] Rosenbaum M. A., Haridas M., McHenry C. R. (2008). Life-threatening neck hematoma complicating thyroid and parathyroid surgery. *American Journal of Surgery*.

[16] Sy A., Ndiaye M., Baldé D. (2012). Les complications de la chirurgie thyroïdienne. A propos de 259 thyroïdectomies réalisées à l’hôpital militaire de Ouakam. *J Afr Chir*.

[17] Kraimps J.-L., Marechaud R., Gineste D. (1993). Analysis and prevention of recurrent goiter. *Surgery Gynecology and Obstetrics*.

[18] Elfenbein D. M., Schneider D. F., Chen H., Sippel R. S. (2014). Surgical site infection after thyroidectomy: A rare but significant complication. *Journal of Surgical Research*.

[19] Dionigi G., Rovera F., Boni L., Dionigi R. (2008). Surveillance of surgical site infections after thyroidectomy in a one-day surgery setting. *International Journal of Surgery*.

[20] Musa A. A., Banjo A., Agboola O., Osinupebi O. (2012). Failure to heal of thyroidectomy wound due to gossypiboma and stitch sinus: Report of two cases. *Journal of Surgical Technique and Case Report*.

[21] Mote D. G. (2014). Gauzoma: A very rare cause of post-thyroidectomy surgical site infection. *International Journal of Biomedical Research*.

[22] Prokopakis E., Kaprana A., Velegrakis S. (2013). Intraoperative recurrent laryngeal nerve monitoring in revision thyroidectomy. *European Archives of Oto-Rhino-Laryngology*.

[23] Renou G., Diore K., Radulescu M. Les reprises chirurgicales en pathologie thyroïdienne. *Fr ORL*.

[24] El-Din Hussain M. S., Mahmoud M. H., Morad M. A. (2000). Morbidity of secondary thyroidectomy in patients with prior thyroid surgery for benign disease. *Suez Canal Univ Med J*.

[25] Pezzullo L., Delrio P., Losito N. S., Caracò C., Mozzillo N. (1997). Post-operative complications after completion thyroidectomy for differentiated thyroid cancer. *European Journal of Surgical Oncology*.

